# Long Non-Coding RNAs in Haematological Malignancies

**DOI:** 10.3390/ijms140815386

**Published:** 2013-07-24

**Authors:** Andoni Garitano-Trojaola, Xabier Agirre, Felipe Prósper, Puri Fortes

**Affiliations:** 1 Laboratory of Myeloproliferative Syndromes, Oncology Area, Foundation for Applied Medical Research, University of Navarra, Pamplona 31008, Spain; E-Mails: agaritano@alumni.unav.es (A.G.-T.); xaguirre@unav.es (X.A.); fprosper@unav.es (F.P.); 2 Hematology Service and Area of Cell Therapy, University of Navarra Clinic, University of Navarra, Pamplona 31008, Spain; 3 Department of Hepatology and Gene Therapy, Foundation for Applied Medical Research, University of Navarra, Pamplona 31008, Spain

**Keywords:** lncRNAs, leukemia, hematologic malignancies

## Abstract

Long non-coding RNAs (lncRNAs) are functional RNAs longer than 200 nucleotides in length. LncRNAs are as diverse as mRNAs and they normally share the same biosynthetic machinery based on RNA polymerase II, splicing and polyadenylation. However, lncRNAs have low coding potential. Compared to mRNAs, lncRNAs are preferentially nuclear, more tissue specific and expressed at lower levels. Most of the lncRNAs described to date modulate the expression of specific genes by guiding chromatin remodelling factors; inducing chromosomal loopings; affecting transcription, splicing, translation or mRNA stability; or serving as scaffolds for the organization of cellular structures. They can function in *cis*, cotranscriptionally, or in *trans*, acting as decoys, scaffolds or guides. These functions seem essential to allow cell differentiation and growth. In fact, many lncRNAs have been shown to exert oncogenic or tumor suppressor properties in several cancers including haematological malignancies. In this review, we summarize what is known about lncRNAs, the mechanisms for their regulation in cancer and their role in leukemogenesis, lymphomagenesis and hematopoiesis. Furthermore, we discuss the potential of lncRNAs in diagnosis, prognosis and therapy in cancer, with special attention to haematological malignancies.

## 1. Introduction

Transcriptome analysis by tiling arrays and RNA sequencing has led to the amazing conclusion that while 70%–90% of the genome is transcribed, only 2% is dedicated to the transcription of protein coding sequences [1]. This result has caused a great impression in a scientific community that is deeply proteocentric, *i.e.*, is dedicated to the study of proteins and generally does not pay much attention to other molecules such as lipids or RNAs.

Most cellular RNA is composed of highly expressed non-coding RNAs whose relevance in cell functionality has been well-known for years. However, their transcription requires a relatively small proportion of the genome. These housekeeping non-coding RNAs include transfer RNAs (tRNAs) and ribosomal RNAs (rRNAs), required for mRNA translation; small nuclear RNAs (snRNAs), essential for splicing; and small nucleolar RNAs (snoRNAs), involved in RNA modification. More recently, several small RNAs have been described as playing essential roles in gene expression and transposon silencing. These include microRNAs (miRNAs), small interfering RNAs (siRNAs) and piwi interacting RNAs (piRNAs). Less clear is the role and the molecular mechanisms involved in the function of other small RNAs derived from retrotransposons or 3′ untranslated regions or associated with transcription start sites, promoters, termini or repeats. All these non-coding RNAs, with the exception of some of the housekeeping RNAs (some rRNAs and a few snRNAs and snoRNAs), share the common characteristic of being smaller than 200 nts. Therefore the remaining non-coding RNAs, longer than 200 nts, have been grouped under the name of long non-coding RNAs (lncRNAs).

LncRNAs have a terrible name. They are not really long, just longer than the limit of 200 nts imposed by small RNAs. In fact, the average size of coding mRNAs is near 2500 nts while the average length of all the lncRNAs recently described by the Encode project is less than 600 nts [2]. Thus, most of the long non-coding RNAs are shorter than the coding mRNAs, even if some of the lncRNAs may be longer than 100 kbs. Apart from not being really long, it is difficult to determine whether lncRNAs are indeed non-coding. Traditionally, lncRNAs have been characterized by what they do not have: they lack open reading frames (ORFs) longer than 100 amino acids, conserved codons and homology to protein databases [3,4]. Therefore, they have poor coding potential, although they could still code for small open reading frames or non-conserved peptides. Some authors have also analyzed coding capacities of specific lncRNAs by matching their sequences with ribosome footprints or peptide fragments from mass spectrometry analysis. Hits would indicate translation [5–8]. In spite of these efforts, it should be borne in mind that what makes lncRNAs interesting for most scientists is not whether they can encode for proteins or not but the fact that they are functional as RNA molecules. The demonstration of function as an RNA should be required for annotation as an lncRNA, as a functional long RNA is the best definition for lncRNAs. To complicate things further, there are several cases of coding mRNAs that contain regulatory RNA elements and act as bifunctional RNAs; on one hand they code for a protein (p53, for instance) and on the other hand they have a function as RNAs [9–14]. Furthermore, several coding genes are transcribed to non-coding alternative splicing variants.

Functional or lncRNA genes are very similar to coding genes at the DNA and chromatin level as they share the same epigenetic marks. Similar to mRNAs, most lncRNAs are transcribed from RNA polymerase II, are capped at the 5′ end, contain introns and approximately 40% are polyadenylated at the 3′ end [15]. The lncRNAs recently described by Encode show a bias for having just one intron and a trend for less-efficient cotranscriptional splicing [8,16]. It has been estimated that there could be as many lncRNA genes as coding genes, but the number of lncRNAs is still growing and some authors consider that it could increase from ~20,000 to ~200,000 [17,18]. Compared to mRNAs, most lncRNAs localize preferentially to the nucleus, are more cell type specific and are expressed at lower levels [19]. In fact, there is less than one copy per cell of many lncRNAs. The low expression levels and the fact that the sequence of lncRNAs is poorly conserved have convinced many scientists that they are not relevant for cell functionality. However, although lncRNAs are under lower selective pressure than protein-coding genes, sequence analysis shows that lncRNAs are under higher selective pressure than ancestral repeat sequences with neutral selection. Moreover, promoters of lncRNAs have similar selection levels than promoters of protein coding genes [8]. Even in the absence of strong sequence conservation, the genomic location and structure of many lncRNAs is conserved together with short stretches of sequences, suggesting that lncRNAs could be under selective pressure to maintain a functional RNA structure rather than a linear sequence [8].

Recent publications in the field have led to the hypothesis that many lncRNAs may be key regulators of development and may play relevant roles in cell homeostasis and proliferation. In fact, several lncRNAs have been described that function as oncogenes or tumor suppressors [20]. It is expected that for cell biology the role of lncRNAs could be as revolutionary as the role of small non-coding RNAs such as miRNAs. miRNA studies have highlighted the relevance of gene regulation in cell homeostasis, differentiation and proliferation and may impact the clinic with new therapies and new diagnostic and prognostic tools for many diseases. The relevance of miRNAs has been clearly established for haematological malignancies [21,22]. In this review we will summarize what is known about lncRNAs in normal haematopoiesis and in haematological tumors. Even though many more studies need to be done, the results obtained thus far suggest that several lncRNAs may be key molecules in haematopoiesis and in the pathogenesis of haematological malignancies.

## 2. Classes of lncRNAs and lncRNA Functionality

### 2.1. Classification by Genomic Location

Under the name of lncRNAs there are RNAs with many different characteristics, which complicates classification. Therefore a well accepted method is based on genomic location rather than on functionality, conservation or origin. From a genetic point of view lncRNAs can be classified into one or more of the following categories: (a) sense, when overlapping with one or more exons of another transcript in the same strand; (b) antisense, when overlapping with one or more exons of another transcript in the opposite strand; (c) intronic, when derived from an intron of another transcript; (d) divergent or bidirectional, when they share a promoter with another transcript in the opposite strand and therefore are coregulated; (e) intergenic, when they are independent, located in between two other genes. Long intergenic non coding RNAs (lincRNAs) are a special class of intergenic lncRNAs whose genes have histone mark signatures of active transcription (trimethylation in lysine 4 and lysine 36 of histone 3: H3K4m3, H3K36m3) [23].

In the case of antisense transcripts, classification based on genomic location helps to predict functionality. 50%–70% of sense transcripts have natural antisense partners (NATs) [24–26]. NATs are generally involved in the regulation in *cis* of the corresponding sense RNA by mechanisms that act at the transcriptional and posttranscriptional level. NATs can induce transcriptional interference or recruit chromatin modifiers and remodelers to establish a local transcriptionally active or inactive chromatin conformation [27]. Posttranscriptionally, examples of NATs exist that regulate imprinting, RNA editing, splicing, by blocking binding of the spliceosome to the 5′ splice site of an intron leading to intron retention [28–32] or translation and stability by forming a duplex with the sense RNA that masks the binding site for miRNAs [33]. Thus, NATs can modify processing and induce or reduce the expression or the translation of their sense counterpart. Some intronic lncRNAs also regulate the expression of their genomic partners. Intronic lncRNAs may be generated by stabilization of the intron after splicing of the host gene but, more commonly, they are produced from independent transcription. Some intronic non-coding RNAs are associated with polycomb-related repressive histone marks along the promoter region and gene body of their host gene, which results in local transcriptional silencing [34].

### 2.2. Classification by Specific Characteristics

Most lncRNAs with special characteristics cannot be easily classified into a single group according to genomic location. These include enhancer RNAs (eRNAs), lncRNA-activating (lncRNA-a) genes, transcribed ultraconserved regions (T-UCRs), pseudogenes, telomere-associated ncRNAs (TERRAs), circular RNAs, *etc.*

eRNAs are transcribed by RNA polymerase II at active enhancer regions, characterized by H3 Lys4 monomethylation or Lys27 acetylation and binding of the regulatory protein p300 [35–39]. eRNAs are not polyadenylated. Many are bidirectional and poorly expressed [38,40], but expression of several eRNAs seems to be tightly regulated [38,39]. Although many eRNAs were thought to be by-products of the presence of RNA pol II in enhancers, recent evidence suggests that some may function to control the expression of neighbouring genes [41]. eRNAs are also important in the formation of the chromosomal loopings that bring enhancers closer to promoters [39], and in the induction of, for example, p53-dependent enhancer activity and transcription [42].

lncRNA-a genes generally transcribe intergenic RNAs which are involved in the expression of neighbouring genes [41]. Thus, downregulation of the lncRNA-a results in downregulation of the neighbour gene. This effect requires expression of the Mediator complex and it has been shown that interaction of the lncRNA-a with Mediator is required for the upregulation of nearby genes [43].

T-UCRs and pseudogenes are lncRNAs that share sequence similarity to other mammalian genomes or other regions of the same genome, respectively. There are 481 UCRs longer than 200 bp that are absolutely conserved between human, rat, and mouse genomes [44]. Most are transcribed or T-UCRs in normal human tissues, both ubiquitously and tissue specifically. The high degree of conservation across species implies that T-UCRs may be essential, but deletion of some of these regions in knockout mice has not been associated with a detectable phenotype [45]. One possible function of some T-UCRs is miRNA control, as many T-UCRs have significant antisense complementarity with particular miRNAs and there is a negative correlation between expression of specific T-UCRs and predicted antisense miRNAs targets [46,47]. In fact, some T-URCs have been shown to be targeted by miRNAs.

Pseudogenes originated from duplication of ancestor or parental coding genes (duplicated pseudogenes) or through retrotransposition of processed RNAs transcribed from ancestor genes (processed pseudogenes). Subsequently, they have lost their coding capacity as a result of the accumulation of mutations. When pseudogenes are expressed, they may regulate the expression and function of their parental gene by several mechanisms [48,49]. For instance, pseudogenes may act as miRNA decoys that lead to increased stability and translation of their parental gene [50–53].

Circular RNAs, newcomers to the RNA list, can also function as RNA decoys [54–56]. It is generally accepted that circular RNAs originate from reverse splicing, where the acceptor splice site located downstream binds to an upstream donor splice site. This causes the circularization of the RNA and a tremendous increase in RNA stability, as circular RNAs lack 5′ or 3′ ends and therefore, are resistant to exonucleases. The increased stability of circular RNAs may lead to long-term functionality by miRNA sequestration [57].

### 2.3. Classification as *cis* or *trans*-Acting Molecules

**Figure 1:**
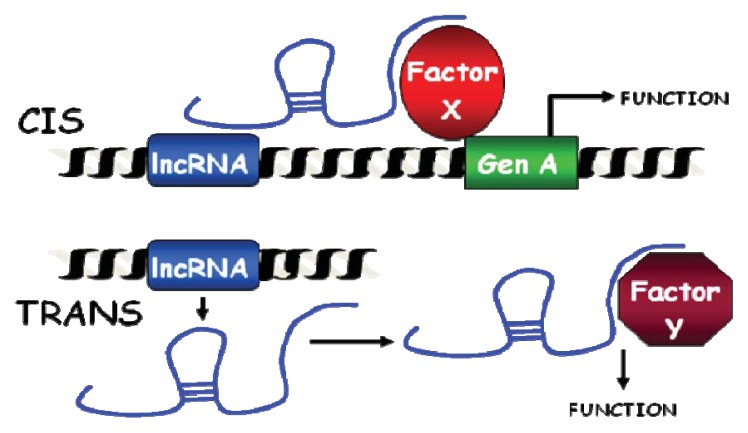
Schematic representation of *cis* and *trans*-acting lncRNAs. *cis*-acting lncRNAs function at the site of transcription and affect the expression of neighbouring genes. *Trans*-acting lncRNAs function away from the site of synthesis.

LncRNAs can also be classified according to their functionality as *cis* and/or *trans* acting molecules (Figure 1). *Trans*-acting lncRNAs function away from the site of synthesis while *cis*-acting lncRNAs function at the site of transcription to affect the expression of neighbouring genes. Several *cis*-acting lncRNAs guide epigenetic regulators to their site of transcription while they are being transcribed. Thus, lncRNA transcription is critical and rapidly creates an anchor to recruit proteins involved in chromatin remodelling [58–61]. This molecular mechanism has tremendous advantages: (i) it responds very fast, as it only requires transcription of an RNA and a proper accumulation of nuclear chromatin remodelers; (ii) it is very specific, as the targeting does not involve RNA-DNA interactions other than those required for lncRNA transcription and (iii) it may function with just a single molecule of lncRNA per locus. This may explain the low abundance of *cis*-acting lncRNAs and the relatively high concentration of lncRNAs close to developmental genes whose expression is strictly controlled [62]. Thus, *cis*-acting lncRNAs control the epigenetic regulation of some imprinted genes. Imprinting depends on the parental origin of the imprinted genes, which play critical roles in mammalian development and therefore, their expression must be tightly regulated [63]. Many imprinted gene loci express lncRNAs that appear to regulate the expression of neighbouring imprinted protein-coding genes in *cis*, allele specifically [64]. The lncRNA *AIR*, for example, silences the neighbouring imprinted genes *SLC22A3*, *SLC22A2* and *IGF2R* [65].

The clear division between *cis* and *trans* acting lncRNAs has been blurred by recent experiments, where exogenously expressed lncRNAs that normally work in *cis*, are able to find their target sites. Thus, even *cis*-acting lncRNAs may have the capacity to act in *trans* [65]. Furthermore, when considering *cis*-acting lncRNAs, the 3D organization of the genome should be taken into consideration. A *cis*-acting lncRNA may control the expression of neighbour genes brought into proximity by chromosome looping.

*Trans*-acting lncRNAs regulate gene expression on a genome-wide scale. A good example is *HOTAIR*, which binds the chromatin-modifying complexes PRC2, LSD1 and CoREST/REST [66–69]. Guiding chromatin remodelers to specific sites is easier to conceive for *cis*-acting lncRNAs. Targeting mediated by *trans*-acting lncRNAs would probably require RNA:DNA:DNA triplex formation via Hoogsteen base-pairing, as has been shown *in vitro* for a promoter-associated lncRNA [70]. However, such interactions may expose the genome to deamination and damage [71,72]. Furthermore, lncRNAs could form secondary and tertiary structures that behave similary to DNA-binding domains from proteins or that bind proteins that mediate DNA binding. This is what has been described for the *XIST* lncRNA, which binds YY1 transcription factor to reach specific sites in the X chromosome [73]. Theoretically, lncRNAs could also form an RNA:DNA hybrid that displaces a single strand of DNA (the so-called R-loop) or an RNA:RNA hybrid of lncRNA with a nascent transcript [74–76].

### 2.4. lncRNA Functionality

Guiding chromatin remodelling factors seems to be the predominant function exerted by lncRNAs. In fact, it has been estimated that 20% of all lncRNAs may bind PRC2 [66]. Several lncRNAs have also been shown to bind to PRC1, the CoREST/REST repressor complex [66], the histone methyltransferase associated with the activating trithorax complex, MLL1 [77,78], and H3-K9 methyltransferase, G9a [65,79]. However, lncRNAs have also been shown to exert several other functions in the cell nucleus and cytoplasm, including regulation of DNA bending and insulation, RNA transcription, splicing, translation and stability, organization of subnuclear structures and protein localization, among others.

DNA looping. CTCF can induce chromosomal bending and protect specific genes from the effects of distal enhancers and regulatory elements. The lncRNA *SRA* can interact with and enhance the function of CTCF [80]. Also, endogenous but not exogenous nascent *HOTTIP* lncRNA, binds target genes via chromosomal looping [81].

Transcription. LncRNAs may activate or inhibit transcription of specific targets. Some lncRNAs act as coactivators that bind transcription factors and enhance their transcriptional activity [82–84]. This is the function of *SRA* lncRNA in the progestin steroid hormone receptor [85,86]. However, some lncRNAs act as decoys of transcription factors [87] and may move them to the cytoplasm to keep them away from their nuclear targets [88]. Thus, p53-induced lncRNA *PANDA* binds transcription factor NF-YA and prevents NF-YA activation of cell death genes [89]. *DHFR* lncRNA forms a triplex structure which sequesters the general transcription factor IIB and prevents transcription of the *DHFR* coding gene [90]. Finally, the act of lncRNA transcription may interfere with transcription initiation, elongation or termination of another sense or antisense gene [91]. Transcriptional interference can also lead to activation of gene expression by inhibiting the action of repressor elements.

Organization of subnuclear structures. LncRNAs can recruit protein factors to nuclear structures. This is the case of lncRNA *MALAT1* and *NEAT-1. MALAT1* recruits serine/arginine–rich splicing factors to nuclear speckles [92]. More importantly, *NEAT-1* is an essential structural component of paraspeckles, subnuclear structure implicated in RNA splicing and editing [93,94]. Depletion of *NEAT-1* leads to loss of paraspeckles while overexpression of *NEAT-1* causes an increase in the number of paraspeckles [95–97]. *MALAT1* and *NEAT-1* are genomic neighbours overexpressed in several tumors compared to healthy tissues. Surprisingly the mouse knockouts of either *NEAT-1* or *MALAT1* had no detectable phenotype, suggesting that there could be redundant or compensatory molecules [98–101].

Splicing. Splicing can be inhibited by lncRNAs antisense to intron sequences that impede spliceosome binding causing intron retention [28–32]. Furthermore, alternative splicing can be altered by lncRNA-mediated sequestration or modification of splicing factors. Thus, *MALAT1* binds splicing factors present in nuclear speckles and modulates the activity of SR proteins, involved in the selection of splice sites, and therefore regulates the splicing of many pre-mRNAs [92]. Some snoRNA-containing lncRNAs (sno-lncRNAs) are retained close to their sites of transcription where the splicing factor Fox2 is enriched. Changes in the level of the sno-lncRNA lead to a nuclear redistribution of Fox2 and to changes in alternative splicing. Thus, the sno-lncRNAs could function as a regulator of splicing in specific subnuclear domains [102].

Translation. LncRNAs have been described that increase or inhibit translation of specific targets [103,104]. Expression of antisense *UCHL1* lncRNA leads to an increase in Uchl1 protein level without any change at the Uchl1 mRNA level. A repetitive SINEB2 sequence is required for this function. Under cap dependent translation inhibition due to stress, *UCHL1* lncRNA moves from the nucleus to the cytoplasm, binds to Uchl1 mRNA and allows its cap-independent translation. Thus, *UCHL1* lncRNA could behave as a mobile internal ribosomal entry sequence.

Stability. LncRNAs have been described that increase or decrease stability of specific targets [105,106]. Binding of lncRNAs containing ancestral Alu repeats to complementary Alu sequences in the 3′UTR of coding mRNAs forms a dsRNA recognized by the dsRNA binding protein Stau1, which induces Stau-mediated RNA decay [106]. Instead, lncRNA *TINCR* localizes to the cytoplasm, where it interacts with Stau1 and promotes the stability of mRNAs containing the TINCR box motif [105].

miRNA binding. LncRNAs can regulate mRNA stability and translation by binding to miRNAs and preventing their action. Besides the already described role of some pseudogenes and circular lncRNAs in miRNA sequestration, other lncRNAs such as *linc-MD1*, have been shown to serve as “sponge” for miRNAs. *Linc-MD*1 binds two miRNAs, which downregulate transcription factors involved in muscle differentiation and therefore muscle differentiation is induced upon *Linc-MD*1 expression [107].

LncRNAs have been implicated in many other different functions. LncRNA *NRON* is a repressor of NFAT by binding β-importins and regulating the nuclear trafficking of NFAT [88]. *TERC* is a well-known telomerase-associated lncRNA that serves as a template for the synthesis of chromosome ends. The dsRNA-protein kinase PKR may be activated by binding to a lncRNA [108]. It is expected that in the near future novel and unexpected mechanisms of lncRNA functionality will be discovered. For instance, to date few lncRNAs have been described to have catalytic properties.

The high number of lncRNAs and their heterogeneity helps them to exert such a myriad of functions. In fact, all lncRNA functions respond to just three different mechanisms: decoys, scaffolds and guides [109]. Decoy-acting lncRNAs impede the access of proteins such as transcription factors and RNAs such as miRNAs to their targets. LncRNAs *MD-1* and *PANDA* act as decoys for miRNAs and transcription factors, respectively [89,107]. Scaffold-acting lncRNAs serve as adaptors to bring two or more factors into discrete ribonucleoproteins (RNPs) [110]. LncRNA *TERC*, *HOTAIR* or *NEAT-1* act as scaffolds to form the telomerase complex [111], a silencing complex [69] or the paraspeckle, respectively [93,94]. Guide-acting lncRNAs are required to localize protein complexes at specific positions. *XIST* or *AIR* lncRNAs act as guides to target gene silencing activity in an allele-specific manner. Guide lncRNAs such as *HOTAIR*, can also behave as scaffolds.

It is conceivable that lncRNAs may function through linear or structured domains. Linear domains may bind proteins but also RNA or, possibly, DNA sequences by perfect (e.g., antisense lncRNAs with their sense counterpart) or imperfect complementarity. Novel linear domains able to bind and regulate mRNAs, miRNAs or other lncRNAs could be very easily created evolutionarily. In many cases though, the secondary and tertiary structure of lncRNAs dictates their function. Thus, lncRNAs generally have complex structures with higher folding energies than those observed in mRNAs [112]. Proteins are expected to be the major partners of lncRNAs to form functional RNP particles. RNA binding proteins represent more than 15% of the total amount of proteins [113]. In several cases studied to date, interaction between proteins and RNAs results in conformational changes to the protein, the RNA or both, which could endow the complex with a novel ability.

LncRNA function impacts cell behaviour. LncRNAs have specially emerged as regulators of development. Some transcription factors involved in pluripotency bind promoter regions of more than 100 mouse lncRNAs [15]. 26 lincRNAs have already been described as being required for the maintenance of pluripotency in mouse [114]. Two lncRNAs regulated by pluripotency transcription factors such as Oct4 and Nanog are essential for pluripotency maintenance, as they, in turn, control the expression of Oct4 and Nanog [115]. Therefore, these lncRNAs participate in positive regulatory loops. Similarly, several lncRNAs have been implicated in human disease, including several cancers [116]. Dysregulated lncRNAs have been described in heart disease, Alzheimer disease, psoriasis, spinocerebellar ataxia and fragile X syndrome [33,117–121] and in several tumours including breast, brain, lung, colorectal, prostate and liver cancers, melanoma, leukaemia and others [46,68,116,122–128]. LncRNAs have been described that function as oncogenes [129], tumour suppressors [23,130] or drivers of metastatic transformation, such as *HOTAIR* in breast cancer [68]. In this review we will concentrate on those lncRNAs whose expression is altered in haematological malignancies.

## 3. LncRNAs Deregulated in Haematological Malignancies

The impact of non-coding RNAs on haematological malignancies has been well described for microRNAs [131,132]. The list of lncRNAs involved in the initiation and progression of blood tumors is still very short and expected to grow exponentially in the near future. Some of the lncRNAs that play a role in haematological malignancies (Table 1) are in fact host genes of miRNAs with oncogenic or tumour suppressor properties. Others endow oncogenic or tumour suppressor properties in the long non-coding RNA molecule. The mechanism of action of few of them has been studied in some detail.

### 3.1. Host Genes of Small RNAs

#### 3.1.1. BIC and C13ORF25

**Figure 2:**
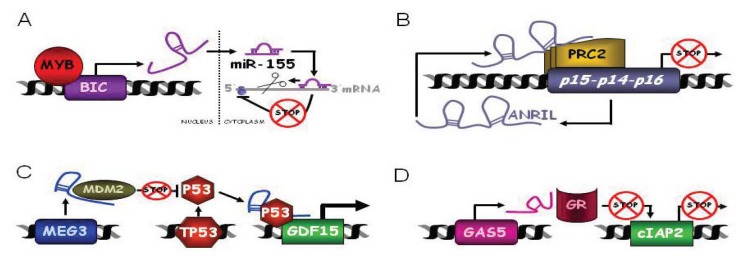
Schematic representation of the function of lncRNAs deregulated in haematological malignancies. (**A**) *BIC*. Myb transcription factor increases the expression of *BIC* in several leukemias and lymphomas. This results in increased levels of *miR-155* and *miR-155-*mediated downregulation of several tumor suppressor genes; (**B**) *ANRIL*. The INK4 *p15INK4b-p14ARF-p16INK4a* cluster transcribes for an antisense transcript named *ANRIL*; PcG complex (PRC2) is targeted to the INK4 locus by *ANRIL*, and locus expression is inhibited; (**C**) *MEG3. MEG3*, among other functions, stimulates p53-dependent tumor suppressor pathways by several mechanisms. *MEG3* down-regulates MDM2 expression, therefore decreasing p53 MDM2-mediated degradation. *MEG3* increases p53 protein levels and stimulates p53-dependent transcription. *MEG3* enhances p53 binding to some target promoters such as *GDF15*; (**D**) *GAS5. GAS5* binds the DNA binding domain of glucocorticoid receptors (GR) and impedes GR binding to DNA and induction of GR-dependent genes such as *cIAP2*.

Some lncRNAs were described to have oncogenic properties in blood cells before the discovery of miRNAs. This is the case of the B cell Integration cluster (*BIC*) or host gene *mir-155* (*MIR155HG*) (Figure 2A). *BIC* and *miR-155* expression is increased in Hodgkin lymphoma, Acute Myeloid Leukemia (AML) and Chronic Lymphocytic Leukemia (CLL) but it is not detected in healthy samples [133]. Increased expression of BIC and *miR-155* results from transcription activation by the MYB transcription factor [134] and leads to *miR-155-*mediated downregulation of several tumor suppressor genes [135]. In this case, the lncRNA *BIC* plays an important role in the regulation of *miR-155* which is directly involved in the lymphomagenesis or leukemogenesis. Similarly, *C13ORF25* or host gene *mir-17* (*MIR17HG*) encodes the *miR-17-92* cluster and its expression is increased in B-cell lymphoma [136], Mantle Cell Lymphoma (MCL) [137] and other tumors [138,139].

#### 3.1.2. nc886 or *vtRNA2-1*

*vtRNA2-1*, previously known as *pre-miR-886*, is a short ncRNA suppressed in a wide range of cancer cells that inhibits activation of protein kinase R (PKR) [140]. Even if nc886 is shorter than 200 nts and therefore is not a lncRNA, its relevance in AML merits a short description. *vtRNA2-1* is transcribed from the long arm of chromosome 5 region whose deletion is associated with poor outcome in AML. Furthermore, decreased expression by monoallelic or biallelic DNA methylation correlates with a worse outcome in AML patients [141]. Thus, *vtRNA2-1* could be a tumour suppressor for AML and its role could be mediated by PKR.

#### 3.1.3. *PVT1*

It is not clear whether the role of Plasmacytoma variant translocation 1 (*PVT1*) lncRNA in haematological malignancies depends exclusively on being a miRNA host gene. The *PVT1* gene is transcribed to several mature RNAs by alternative splicing, including a cluster of seven miRNAs, six of them annotated in the miRBase as *miR-1204*, *miR-1205*, *miR-1206*, *miR-1207-5p*, *miR-1207-3p*, and *miR-1208*. The function of these miRNAs is unknown with the exception of *miR-1204. miR-1204* has been involved in different roles related to development, differentiation and senescence [146,169]. On one hand *miR-1204* has been described as increasing p53 levels and causing cell death [148]. In fact *PVT1* expression is induced in response to p53 [148]. On the other hand, *miR-1204* has been shown to activate Myc and cell proliferation in mouse pre- B cell lines [146,147].

*PVT1* is located in chromosome region 8q24.21, relatively close to the transcription factor c-Myc. Translocations within c-Myc or *PVT1*, which cause the overexpression of these two oncogenes compared to healthy cells, are characteristics associated with B cell malignancies including Burkitt Lymphoma (BL), AIDs, Non-Hodgkin lymphoma, mouse plasmacytoma (Pct) and multiple myeloma (MM) [147]. Furthermore, *PVT1* is in a susceptibility locus for classical Hodgkin’s lymphoma [145] and a SNP that causes increased *PVT1* expression is associated with prostate cancer risk [170]. *PVT1* is overexpressed, compared to healthy tissues, in breast and ovarian cancer, pediatric malignant astrocytomas, AML and Hodgkin lymphoma [171], suggesting that *PVT1* could be an oncogene. In fact, upregulation of *PVT1* contributes to tumor survival and chemoresistance [171–174] while its downregulation inhibits cell proliferation and induces a strong apoptotic response [171]. It has been proposed that *PVT1* regulates c-Myc expression but also that *PVT1* is regulated by c-Myc [175]. However, some authors suggest that Myc and *PVT1* contribute to cancer by different mechanisms [147,171]. Further studies are required to understand the role of *PVT1* in tumorigenesis and to determine whether the miRNAs encoded by *PVT1* mediate its functionality.

### 3.2. LncRNAs with Oncogenic Properties

#### *ANRIL* or *CDKN2B-AS1*

Antisense Non-coding RNA in the INK4 Locus (*ANRIL*) or *CDKN2B-AS1* is transcribed antisense to the *p15INK4b-p14ARF-p16INK4a* cluster, whose members are key effectors of oncogene-induced senescence (Figure 2B). The INK4 proteins are induced during aging and in premalignant lesions, limiting tumor progression. Therefore, expression of the *INK4b-ARF-INK4a* locus is tightly controlled and the Polycomb group (PcG) complexes are required to initiate and maintain silencing of this locus [176,177]. PcG complexes are targeted to the locus by *ANRIL* [178]. Depletion of *ANRIL* disrupts binding of the PRC2 component SUZ12 to the locus, increases the expression of p15INK4b and inhibits cellular proliferation. *ANRIL*, as a pol II nascent transcript, also controls cellular lifespan by targeting the PRC1 component CBX7 to the INK4 locus [27].

Genome-wide association studies revealed that *ANRIL* is located in a genetic susceptibility locus (9p21) associated with several diseases, including coronary artery disease (CAD), atherosclerosis, intracranial aneurysm, type 2 diabetes, and several cancers, such as glioma, basal cell carcinoma, nasopharyngeal carcinoma, and breast cancer [179]. Several single nucleotide polymorphisms (SNP) in this locus alter *ANRIL* structure [180] and *ANRIL* gene expression [181,182], mediating susceptibility to disease. There is a statistically significant association between an *ANRIL* polymorphism and Philadelphia positive Acute Lymphoblastic Leukemia (Ph+ ALL) [183]. Furthermore, 69% of samples (*n* = 16) from patients with ALL and AML showed relatively increased expression of *ANRIL* and downregulated p15 compared to controls [130]. The expression of *ANRIL*, CBX7, and EZH2 is coordinated and elevated in preneoplastic and neoplastic tissues, leading to decreased p16INK4a expression and decreased senescence [27]. In fact, the *INK4b-ARF-INK4a* locus is subject to frequent deletion or hypermethylation in cancers, including leukemia, melanoma, lung and bladder cancers [177].

### 3.3. LncRNAs with Tumor Suppressor Properties

#### 3.3.1. *MEG3*

The maternally expressed gene 3 (*MEG3*) was the first lncRNA proposed to function as a tumor suppressor (Figure 2C). *MEG3* is a paternally imprinted polyadenylated RNA, expressed in many normal human tissues as several alternative splicing variants [184,185]. *MEG3* expression was decreased compared to healthy tissues in various brain cancers (pituitary adenomas, glioma and the majority of meningiomas and meningioma cell lines) [149,154], bladder, lung, renal, breast, cervix, colon and prostate cancers and haematological malignancies such as MM, AML or myelodysplastic syndromes. Surprisingly *MEG3* is overexpressed in Wilms tumor and may be increased or decreased in different hepatocellular carcinomas *versus* healthy livers [186].

The last intron of *MEG3* lncRNA encodes the evolutionarily conserved *miR-770* [187] and *MEG3* isoforms can contain several small open reading frames that are not required for *MEG3* function [152,153]. Instead, the *MEG3* secondary structure, rather than primary sequence, is critical to maintaining function [152]. *MEG3* lncRNA localizes to the nucleus, although some cytoplasmic *MEG3* transcripts have been detected [184,188,189]. In the nucleus, *MEG3* binds to PRC2 to control the imprinting of the *DLK1* locus, where *MEG3* belongs. Furthermore, *MEG3* stimulates both p53-dependent and p53-independent tumor suppressor pathways [149,150,152–155]. *MEG3* activates the tumor suppressor protein p53 at different levels. On one hand *MEG3* down-regulates MDM2 expression, therefore decreasing p53 MDM2-mediated degradation [150]. On the other hand, *MEG3* significantly increases p53 protein levels and stimulates p53-dependent transcription [155]. Finally, *MEG3* enhances p53 binding to some target promoters such as *GDF15* [152,153]. Ectopic expression of *MEG3* RNA leads to p53 accumulation and inhibition of cellular proliferation [153,185]. Inactivation of *MEG3* in the brain increases the expression of genes involved in angiogenesis, suggesting that the tumour suppressor function of *MEG3* works, in part, by inhibiting angiogenesis [190]. In bladder cancer a negative correlation has been shown between *MEG3* expression and autophagy [191].

#### 3.3.2. *DLEU1* and *DLEU2*

Deleted in leukemia 1 (*DLEU1*) and 2 (*DLEU2*) are two genes transcribed head to head in a 30-kb region located in the long arm of chrormosome 13 (13q14), which is lost in more than 50% of patients with CLL and that predicts a poor prognosis [192]. The homozygous loss of this region has great effects on the regulation and control of normal CD5+ B lymphocytes and their homeostasis. Recent studies show that *DLEU1* and *DLEU2* control transcription of their neighbouring candidate tumour suppressor genes, which may act as positive regulators of NF-kB activity [156]. As binding of *DLEU1* and *DLEU2* to chromatin has not been detected, it has been proposed that they regulate neighbouring gene expression by divergent transcription. In addition, the intron 4 of *DLEU2* encodes the miRNAs *hsa-miR-16-1* and *hsa-miR-15a.* This miRNA cluster exerts a crucial role in the tumorigenesis of CLL, in part, regulating the oncogene *BCL2* [193]. Knocking out *hsa-miR-16-1* and *hsa-miR-15a* in mice leads to a lymphoproliferative disease [194]. However the knockout model of *DLEU2*, which includes deletion of *hsa-miR-16-1* and *hsa-miR-15a* as well, shows a more aggressive phenotype than the *hsa-miR-16-1*/*hsa-miR-15a 6* knockout model alone, suggesting that *DLEU2* can participate in CLL development on its own. In fact, increased expression of *DLEU2* leads to reduced proliferation and clonogenicity [195].

#### 3.3.3. *GAS5*

Growth arrest specific 5 (*GAS5*) is induced under starvation conditions and is highly expressed in cells that have arrested growth [196,197]. *GAS5* modulates cell survival and metabolism by antagonizing the glucocorticoid receptor (GR) [87] (Figure 2D). *GAS5* binds the DNA binding domain of GRs directly, preventing GRs from binding to DNA, from functioning as transcription activators and from reducing cell metabolism [87]. *GAS5* could regulate other receptors (androgen, mineralocorticoid and progesterone but not estrogen receptors) by the same means [87]. Expression of *GAS5* is sufficient to repress GR-induced genes, such as the cellular inhibitor of apoptosis 2 (*cIAP2*) and sensitizes cells to apoptosis [87]. Thus, *GAS5* behaves as a tumor suppressor. *GAS5* expression is decreased in breast cancer and is almost undetectable in growing leukemia cells and increases after density-induced cell cycle arrest [87,196,197]. At the same time, *GAS5* has been shown to be regulated by the mammalian target of rapamycin (mTOR) pathway and to mediate the effect of rapamycin on the cell cycle in T cells [198]. Downregulation of *GAS5* by RNA interference protects leukemic and primary human T cells from the anti-proliferative effect of rapamycin [199].

### 3.4. LncRNAs with Dual Functions

#### 3.4.1. *H19*

*H19* is an imprinted lncRNA located close to the *IGF2* gene. *H19* is expressed form the maternal allele and *IGF2* from the paternal allele [59,200]. A key feature of cancer is the loss of this imprinting, which results in the well documented overexpression of *H19* in cancers of the colon, liver, breast and bladder and in hepatic metastases, compared to healthy tissues [200–204]. Loss of *H19* imprinting has been described in adult T-cell leukaemia/lymphoma (ATL) [157] and decreased *H19* expression was found in the bone marrow of patients with clinically untreated chronic myeloproliferative disorders, including chronic myeloid leukemia (CML), polycythemia vera (PV), essential thrombocythemia (ET), primary myelofibrosis (PMF) and chronic myelomonocytic leukaemia (CMML) [205,206] and AML [207].

*H19* can behave as an oncogene or as a tumour suppressor [59]. *H19* expression can be activated by the oncogene c-Myc [200] and downregulated by the tumour suppressor p53 [208,209]. Downregulation of *H19* by RNAi blocks cell growth and clonogenicity of lung cancer cell lines [200] and decreases xenograft tumour growth of a hepatocellular carcinoma cell line [203]. Furthermore, *H19* is the precursor of miR-675, which downregulates the tumor suppressor retinoblastoma in human colorectal cancer [210]. All these results indicate that *H19* is an oncogene [210]. However, depletion of *H19* caused increased polyp count in a mouse model for colorectal cancer [211], larger tumor growth in a mouse teratocarcinoma model and an earlier development of tumours in a mouse hepatocarcinoma model [212]. This dual role as oncogene or tumour suppressor may depend on the cellular environment of the tumour type.

#### 3.4.2. T-UCRs

The expression of many T-UCRs has been described to be significantly altered in tumours such as CLL, colorectal and hepatocellular carcinomas and neuroblastomas [46,162,213,214]. Certain SNPs in T-UCR genes were associated with increased familial breast cancer risk [163]. Moreover, T-UCR transcription profiles can be used to differentiate types of human cancers and predict patient outcome [213]. Some T-UCRs seem tumour specific, such as *UC.73A* and *UC.338*, which are decreased in colon cancer [215]. In fact, some T-UCRs differentially expressed in a particular human cancer locate in fragile sites or cancer-associated genomic regions specifically associated with that type of cancer [216]. This is the case of *UC.349A* and *UC.352*, differentially expressed between normal and leukemic CD5-positive cells [46] and located within a chromosomal region linked to susceptibility to familial CLL [217]. Moreover, a profile of 19 T-UCRs (8 up- and 11 down-regulated) was able to differentiate between normal, CLL, colorectal, and hepatocarcinoma samples. Expression of five T-UCRs was able to divide a CLL cohort into two prognostic groups [46]. Expression of these diagnostic T-UCRs negatively correlated with a previously defined CLL miRNA signature, suggesting a mechanism for miRNA regulation of these T-UCRs [218].

### 3.5. LncRNAs Poorly Characterized in Haematological Malignancies

***LincRNA-p21***: is a p53 activated lncRNA identified in mouse that binds to and guides hnRNP K to target genes. *LincRNA-p21* bound hnRNP K acts as a transcriptional repressor that leads to the induction of apoptosis [23]. As BCR-ABL1 stimulates hnRNP-K expression and stability and promotes tumor progression, it has been suggested that *lincRNA-p21* could play a relevant role in acute or chronic leukemia [219,220]. Furthermore, *lincRNA-p21* can inhibit the translation of target mRNAs [104]. In the absence of HuR, *lincRNA-p21* is stable and interacts with the mRNAs *CTNNB1*, *JUNB* and translational repressor Rck, repressing the translation of the targeted mRNAs [104].

***TCL6:*** T cell Leukemia/Lymphoma 6 (*TCL6*) is transcribed from a locus involved in translocations and inversions with T cell receptor (*TCR*) [221]. These rearrangements in TCR commonly lead to activation of *TCL6* lncRNA and other oncogenes related to T cell leukemogenesis [151].

***WT1-AS***: is an antisense lncRNA to WT-1, a well-characterized developmental gene that is mutated in Wilms’ tumor (WT) and AML. *WT1-AS* has been shown to regulate WT1 protein levels. *WT1-AS* binds the exon 1 of WT1 mRNA in the cytoplasm. It has been suggested that the abnormal splicing of *WT1-AS* in AML could play a role in the development of this malignancy [159].

***CRNDE***: is overexpressed, compared to healthy tissue, in more than 90% of colorectal adenomas tested, but also in hepatocellular, prostate, brain, kidney and pancreas carcinomas and different haematological neoplasia such as AML, MM and T cell leukemia [160]. *CRNDE* has been described as downregulated in ovarian cancer and tends to be overexpressed in non-differentiated tissues *versus* differentiated controls [160]. *CRNDE* binds PRC2 and the downregulation of *CRNDE* causes upregulation of PRC2 regulated genes, decreases growth and increases apoptosis [66].

***RMRP:*** Ribonuclease mitochondrial RNA processing (*RMRP)* is a lncRNA mutated in Cartilage-Hair Hypoplasia (CHH), an autosomal recessive chondrodysplasia with short stature, which entails a high risk of developing Non-Hodgkin lymphoma disease [161,222].

***SNHG5:*** is a precurssor of snoRNAs, similar to *GAS5*, located at the breakpoint of the chromosomal translocation t(3;6)(q27;q15), involved in diffuse large B-cell lymphoma [223].

***HOXA-AS2:****HOXA Cluster Antisense RNA 2* (*HOXA-AS2*) lncRNA is antisense to *HOX3* and *HOX4* coding genes. In an acute promyelocytic leukemia (APL) cell line, *HOXA-AS2* upregulation correlated with inhibition of apoptosis. Treatment with all-*trans* retinoic acid (ATRA) blocked the expression of *HOXA-AS2* and increased apoptosis of the APL cell line [224].

## 4. LncRNAs Involved in Hematopoiesis

The best studied lncRNA in hematopoiesis is *HOTAIRM1* (HOX antisense intergenic RNA myeloid 1). *HOTAIRM1* is as an essential regulator of myeloid cell differentiation that locates at the 3′ end of the *HOXA* cluster and controls *HOXA1* expression [164]. HOXA genes are important transcriptional regulators in normal and malignant hematopoiesis and are known to be important for many cancers including leukemias harbouring MLL rearrangements. *HOTAIRM1* is expressed specifically in the myeloid lineage and is induced during the retinoic acid-driven granulocytic differentiation of the NB4 promyelocytic leukaemia cell line and normal human hematopoietic cells. Knockdown of *HOTAIRM1* affects retinoic acid-induced expression of *HOXA1* and *HOXA4* (but not distal *HOXA* genes) and attenuates induction of myeloid differentiation genes [164].

Other lncRNAs involved in hematopoiesis have also been described. *EGO* (or *EGOT* in human) lncRNA was identified in mouse eosinophil differentiation of CD34+HSCs where it stimulated major basic protein and eosinophil-derived neurotoxin mRNA expression [165]. The lncRNA *PU.1-AS* is an antisense transcript of *PU.1* that negatively regulates *PU.1* mRNA translation by a mechanism similar to miRNAs [166]. *PU.1* is a master hematopoietic transcriptional regulator essential for normal hematopoietic development and suppression of leukaemia development. LincRNA erythroid prosurvival (*EPS*) is one of the about 400 lncRNAs whose expression is modulated during red blood cell formation and is required for differentiation during hematopoiesis in mouse [164,165,167]. *EPS* is an erythroid-specific lncRNA that represses expression of *PYCARD*, a proapoptotic gene, and therefore inhibits apoptosis [167,225]. EPS is not well conserved among mammals. It is presently unclear whether a human version of EPS exists. Finally, *THY-ncR1* is a thymus-specific lncRNA expressed in cell lines derived from stage III immature T cells in which the neighbouring *CD1* gene cluster is also specifically activated [168].

## 5. Regulation of the Expression of lncRNAs Involved in Haematological Malignancies

Altered expression of lncRNAs, similar to that of coding genes, can be the result of genomic alterations, epigenetic regulation or a change in response to transcription factors or stability effectors such as miRNAs.

The presence of mutations in the lncRNA primary sequence correlates highly with human diseases. In fact, most mutations in the genome occur in noncoding regions [226]. Mutations can be large or small. Large-scale mutations are deletions and amplifications of hundreds of nucleotides and chromosomal translocations occurring at fragile sites. Genome-wide analyses looking for fragile sites in lncRNA genes have not yet been performed. However, it is expected that lncRNAs will have a clear association with common chromosomal aberrations similar to that found for miRNAs in human haematological malignancies and carcinomas [46]. In fact, several studies have described lncRNAs affected by large scale mutations. One of the best examples is *ANRIL*, affected by a large germline deletion that includes the complete INK4/ARF locus. This deletion is associated with hereditary cutaneous malignant melanoma and neural system tumors syndrome [179]. *DLEU1* and *DLEU2* lncRNAs also locate in a region commonly deleted in CLL (see above).

Small scale mutations are deletions or insertions of a few nucleotides. The relevance of small scale mutations for lncRNAs is obscured by the fact that little is known about the relevance of the primary sequence in lncRNA functionality and expression. It is expected that small mutations can lead to disease if they affect relevant linear sequences or they alter the structure of domains important in lncRNA functionality or accumulation. In fact, several disease-associated SNPs have been described as affecting the structure of the 5′ and 3′ non-translated regions of coding genes [226]. Furthermore, GWAS studies have shown that SNPs in noncoding regions are associated with higher susceptibility to diverse diseases. Germline and somatic mutations in lncRNA genes have been identified in haematological malignancies and colorectal cancers [227]. SNPs that may affect *ANRIL* have been associated with increased risk of type 2 diabetes and increased susceptibility to coronary artery disease and atherosclerosis [228,229]. Some of these mutations did not affect *ANRIL* transcription or stability. Instead, they disrupt *ANRIL* splicing, resulting in a circular transcript, affecting normal *ANRIL* function and influencing *INK4/ARF* locus expression [180]. Moreover, genetic aberrations of the *GAS5* locus have been found in melanoma, breast and prostate cancers [230–232].

Several lncRNAs are regulated at the transcriptional level. Thus, lncRNAs, such as *lincRNA-P21*, are activated in response to DNA damage by the direct binding of the tumour-suppressor protein p53 to the promoter [23]. Similarly, the expression of several lincRNAs responds to pluripotency factors or oncogenes.

Epigenetic modifications are key regulators of lncRNA expression. This has been well described for *MEG3* and *DLEU1/DLEU2*. Expression of the *MEG3* locus is regulated by two regions, which are hypermethylated in several solid tumours leading to downregulation of *MEG3* expression [185,233,234]. AML patients with aberrant hypermethylation of the *MEG3* promoter showed decreased overall survival [235,236]. Thus, *MEG3* methylation status may serve as a useful biomarker in this leukemia. A similar *MEG3* hypermethylation was observed in 35% of the patients with myelodysplastic syndrome, but in this case there was no statistically significant correlation between *MEG3* hypermethylation and prognosis [235]. Similarly, conserved CpG islands at the transcriptional start sites of *DLEU1* and *DLEU2* were found to be significantly demethylated in a cohort of 143 patients with CLL [156]. Demethylation correlated with transcriptional deregulation of the neighbouring candidate tumour suppressor genes. T-UCRs expression has also been shown to be repressed by CpG island hypermethylation [47,213].

Finally, the expression of lncRNAs can be regulated by miRNAs. Several miRNAs have been described as regulating T-URC expression. This has been best described for *miR-155*, which is overexpressed in CLL compared to healthy cells. *miR-155* targets T-UCRs both *in vitro* and in CLL patient samples [46]. Interestingly, *miR-29a* has also been shown to regulate *MEG3* expression in hepatocarcinoma cell lines [186].

## 6. Concluding Remarks

The identification of lncRNAs and the functional relevance of the lncRNAs studied so far has changed the view about genomes, transcriptomes and gene expression regulation. As the lncRNA field is in its infancy, surprising results are still expected, but a tremendous amount of work needs to be done. Firstly, a systematic identification and annotation of lncRNAs and their expression patterns should be performed and made publically available. As most lncRNAs are tissue specific, all tissues should be profiled. Also, as there is poor sequence conservation between lncRNAs of different species, efforts should be devoted to describing a collection of lncRNAs in different species, including human, mouse, rat, zebra fish, fly, *Arabidopsis* and yeast. As the regulation of expression of lncRNAs is tightly controlled, lncRNAs should also be described in cells responding to different stimuli and in diseased cells. These studies will be complicated further by the fact that lncRNA genes may be transcribed to different transcripts by alternative splicing, polyadenylation and the use of different promoters. It is also necessary to develop a new universal nomenclature that would facilitate routine work with these non coding RNA molecules.

Secondly, functional studies should be performed. Gain and loss of function studies could be carried out to analyze the impact of the lncRNA on the cell phenotype. Transcriptome analysis coupled with gain and loss of function studies could provide clues regarding the cellular pathways affected by the lncRNA, especially if the lncRNA of interest is a regulator of the expression of specific genes. Analysis of lncRNA subcellular localization can also give clues to lncRNA functionality. This can be done with Fish-like techniques that use several labelled oligos at a time. This is essential to detect the expression of lncRNAs, which are generally very structured and not very abundant. The functional domains of lncRNAs should be identified and it should be ruled out that lncRNAs function through the translation of short peptides. Furthermore, it would be desirable to determine the structure of key domains in lncRNAs similarly to what has been done with proteins. This is a major task as there are no reliable methods to determine the secondary structures of lncRNAs with bioinformatic tools. Chemical probing and point mutation studies have been used to determine the structure of many RNAs, but these techniques are very time consuming. Faster results could be obtained by parallel analysis of RNA structure (PARS-Seq) or Frag-Seq, which uses deep sequencing of RNA fragments obtained from RNAs treated with specific RNases that cleave RNA at highly selective structural positions [237]. Furthermore, it would be interesting to identify the factors that bind to relevant lncRNAs. Ideally, specific RNAs should be immunoprecipitated and subjected to mass spectrometry to identify RNA binding proteins. This is not easy, but has been done successfully with pools of cellular RNAs purified by binding to oligodT beads [238]. Theoretically, a lncRNA of interest could be labelled with a domain targeted by a specific protein and the complex could be purified with antibodies specific to the protein. Alternatively, the lncRNA could be immunoprecipitated from cell extracts using biotinylated tiling oligos and streptavidin. Then, lncRNA bound DNA or RNA can be sequenced from the immunoprecipitates. When looking for DNA interactors, this technique has been named Chromatin Isolation by RNA Purification (ChIRP) and has allowed the identification of the natural regions of chromatin that interact with a given lncRNA [58]. Finally, the lncRNA can be transcribed and labelled *in vitro*, incubated with cell extracts and immunoprecipitated with label binding factors.

Finally, detailed analysis of functional lncRNAs will most probably reveal interesting cellular pathways and help to design the architecture of biological tools that may be of interest for biotechnological development. Domains of lncRNAs that function as decoys for miRNAs or transcription factors, mimicking *GAS5* function [239], could be expressed to obtain therapeutic effects. Several lncRNA domains with a specific tertiary structure and a given function could probably be combined to generate lncRNAs with novel functions that could be of therapeutic interest. For instance, an RNA domain involved in the binding to a specific region of the chromatin could be fused to an RNA domain that interacts with factors that silence or activate gene expression or that induce chromosome bendings or genome reorganizations at the specific position. This could be used for silencing of oncogenes or reactivation of tumour suppressor genes. Thus, analysis of the function of lncRNAs is expected to have a tremendous impact on the management of human disease.

Furthermore, strong associations between some lncRNAs and some human diseases have been described. The number of lncRNAs relevant to human diseases is expected to increase as a result of the systematic identification of lncRNAs whose expression is altered in healthy and diseased cells and by genome-wide association studies. In fact GWAS analysis has identified *ANRIL* as a lncRNA involved in atherosclerosis, coronary artery disease, and type 2 diabetes [179]. In the case of cancer and specifically of haematological malignancies, GWAS results at lncRNA loci may identify patient populations at risk of cancer, may classify patients into aggressive or mild cancer groups and may predict a patient’s response to a given therapy [240,241]. Once lncRNAs related to a disease are described, the issue should be addressed whether they are useful signatures for early disease detection, for prognosis or can be used as candidate drug targets for disease intervention [242].

lncRNAs may have specific advantages when used as diagnostic biomarkers, as some show tissue-specific and cancer-specific expression patterns [243]. This is the case of *HULC*, a liver-specific lncRNA highly expressed in primary liver tumours and hepatic metastases of colorectal carcinoma, but not found in primary colon cancers or in non-liver metastases [244,245]. Thus, the expression of *HULC* and other lncRNAs can be used to differentiate between subtypes of the same cancer or to identify unknown primary tumours. Similarly, *PCGEM1*, *PCA3* or *PRNCR1* are three lncRNAs exclusively associated with prostate cancer [123,246,247]. Also, as in the case with miRNAs, some lncRNAs can be detected in body fluids by quantitative reverse transcriptase polymerase chain reaction and therefore enable non-invasive diagnoses. In fact, *HULC* can be detected in the blood of hepatocellular carcinoma patients using qRT-PCR [245]. The ProgensaTM PCA3 urine test, a kit to detect *PCA3* in urine samples from patients with prostate cancer is already being clinically used [248,249]. This specific test can help patients who had a first negative prostate biopsy to avoid unnecessary repeated biopsies [250]. In spite of this fast clinical translation for *PCA3* analysis in prostate cancer, the biological function of *PCA3* is unknown.

LncRNAs can also be used as predictive markers, as lncRNA expression can correlate with patient outcome or response to chemotherapy. Thus, the expression of *HOTAIR* correlates with metastasis and poor outcome in primary breast tumors, gastrointestinal, hepatocellular and colorectal cancers and the expression of *MALAT* correlates with survival in early-stage lung adenocarcinoma [68,124,251–253]. Also, the expression of *XIST* correlates with disease-free survival of Taxol-treated cancer patients [254].

Finally, lncRNAs could be used therapeutically. In cancer, expression of tumour suppressor lncRNAs, such as *GAS5* or *MEG3*, should decrease tumour growth. When the downregulation of tumour suppressor lncRNAs results from aberrant epigenetic mechanisms such as DNA hypermethylation or loss of histone acetylation, demethylating agents or histone deacetylase inhibitors could help to reestablish expression. Otherwise, expression of lncRNAs may require gene therapy delivery systems with viral vectors, which are not efficient in targeting all cells within a tumour. Furthermore, RNA interference can be used to decrease the expression of lncRNAs with oncogenic properties. While many lncRNAs have been silenced using siRNAs, it is generally believed that the secondary structure of lncRNAs hinders siRNA functionality. Instead, expression of lncRNAs with oncogenic or tumour suppressor molecules could be altered with small molecules that affect their promoters. Small molecules, aptamers or stable antisense oligonucleotides could also be identified that target essential structures for oncogenic lncRNA functionality. Thus, preventing the interactions of *HOTAIR* with PRC2, for example, may limit the metastatic potential of breast cancer cells [255]. Even if all these strategies are possible, much investment in this field will be required to transfer lncRNA research to clinical oncology.

## Figures and Tables

**Table 1:** lncRNAs in hematopoiesis and hematological malignancies.

*LncRNAs*	LOCATION	HEMATOLOGIC DISEASE/SYSTEM	FUNCTION	MOLECULAR MECHANISM	MECHANISMS INVOLVED IN DYSREGULATION	CITATIONS
***MIR155HG BIC***	21q21.3	Burkitt, Hodgkin lymphoma, AML, CLL	Host of miRNAs	miR-155	Target MYB and NFKB	[134]
***MIR17HG***	13q31.3	B-cell lymphoma, MCL	Host of miRNAs	miR-17-92	Target MYC	[136,137,142]
***vtRNA2-1***	5q31.1	AML (poor prognosis)		PKR inhibition	DNA methylation Deletion 5q	[140,141]
***PVT1***	8q24.21	MM, Burkitt Lymphoma, T-cell Leukemia, CLL	Oncogene and host of miRNAs	miR-1204 MYC activation	Translocation t(8;14)(q24;q11) t(2;8)(p11;q24) t(8;22)(q24;q11)	[143–148]
***CDKN2BAS1/ ANRIL***	9p21.3	AML, ALL	Oncogene	PRC1 and PRC2 targeting	rs3731217-G SNP Deletion, hypermethylation	[26,128,149–151]
***MEG3***	14q32.2	AML, MM	Tumor suppressor	PRC2 binding to control DLK1 imprinting. p53 activation.	DNA methylation	[149,150,152–155]
***DLEU1/DLEU2***	13q14.2	CLL, MM, Lymphoma	Tumor suppressor	*hsa-miR-16-1* and *15a* BCL2 targeting. NFKB activation	Histone modification, DNA methylation, deletion	[156]
***GAS5***	1q25.1	B-cell Lymphoma, Leukemia	Tumor suppressor	Glucorticoid receptor repression. Regulated by mTOR pathway.	Translocation (1;3)(q25;q27)	[87]
***H19***	11p15.5	AML, CML, MPN, T-cell Leukemia, Lymphoma	Oncogene/tumor suppressor	Activated by Myc and down-regulated by p53. miR-675 targeting Rb	Loss of imprinting	[157]
***T-UCRs***		CLL (prognosis marker)	Oncogene/tumor suppressor	miR control		[46]
***lincRNA-p21***	Not annotated in human	ALL, CML	Tumor suppressor	Activated by p53 binds hnRNP K to induce apoptosis	Not known	[158]
***TCL-6***	14q32.13	T cell leukemia	Poorly characterized	Not described	Translocation and inversions with TCR	[151]
***WT1-AS***	11p13	AML, ALL	Poorly characterized	WT-1 control	Not known	[159]
***CRNDE***	16q12.2	AML, MM, T-cell leukemia	Oncogene	PRC2 and COREST binding	Not known	[160]
***RMRP***	9p13.3	Non-Hodgkin lymphoma	Poorly characterized	Not described	Mutation	[161]
***SNHG5***	6q14.3	B-cell Lymphoma	Poorly characterized	snoRNA host	Translocation (1;3)(q25;q27)	[162]
***HOXA-AS2***	7p15.2	APL	Poorly characterized		Not known	[163]
***HOTAIRM1***	7p15.2	Hematopoietic regulator	Regulator of myelopoiesis	HOX A genes.		[164]
***EGOT***	3p26.1	Hematopoietic regulator	Regulator of eosinophil development			[165]
***PU.1-AS***	11p11.2 Non annotated	Hematopoietic regulator	*PU.1-AS* regulate the hematopoiesis regulator PU.1	PU.1 control		[166]
***EPS***	Mouse 4qC7	Hematopoietic regulator	Regulator of erytropoyesis	Pycard repression		[167]
***ThyncR1***	1q23.1	Hematopoietic regulator	Regulator of T cell selection and maduration.	Riboregulator		[168]
